# Digital Health Technology Use Across Socioeconomic Groups Prior to and During the COVID-19 Pandemic: Panel Study

**DOI:** 10.2196/55384

**Published:** 2024-09-13

**Authors:** Inge Tuitert, Jesse D Marinus, Jelle R Dalenberg, Job TB van 't Veer

**Affiliations:** 1 Academy of Health & Social Studies NHL Stenden University of Applied Sciences Leeuwarden Netherlands; 2 Department of Neurology University Medical Center Groningen Groningen Netherlands; 3 Expertise Center Movement Disorders Groningen University Medical Center Groningen Groningen Netherlands; 4 Planbureau Fryslân Leeuwarden Netherlands; 5 Campus Fryslân University of Groningen Leeuwarden Netherlands

**Keywords:** digital divide, vulnerable groups, digital health apps, adoption, socioeconomics, health technology, digital health, longitudinal, surveys, technology use

## Abstract

**Background:**

Digital technologies have become more important in the health care sector in the past decades. This transition from conventional to digital health care has been accelerated by the impact of the COVID-19 pandemic, which poses the risk of creating a “digital divide,” inadvertently placing those who are older, economically disadvantaged, and have a lower level of education at a disadvantage.

**Objective:**

This study focuses on the influence of socioeconomic factors on the adoption of digital health technology in the Frisian population and how this relation is affected by the COVID-19 pandemic.

**Methods:**

In 2019 and 2020, a panel study was conducted on digital health in the Frisian population in the Netherlands. In the survey, the use of digital health technology was operationalized in a broad sense, going beyond the care context by also including preventative health-promoting solutions generally available on the consumer market, such as wearables and lifestyle apps. First, to assess the influence of socioeconomic factors on the total use of digital health apps, a generalized linear model was fitted with use of digital health app as the dependent variable and socioeconomic factors as between-subject factors on the 2019 data. Second, to analyze whether the use of separate health apps increased from 2019 to 2020, we conducted chi-square tests on different digital health app types. Third, to examine the influence of COVID-19 on the use of digital health apps, a generalized linear mixed model was fitted with the use of digital health apps as the dependent variable, COVID-19 as the within-subject variable, and socioeconomic factors as between-subject factors.

**Results:**

The results indicated that prior to the COVID-19 pandemic, digital health technology use was higher in women, younger people, and those who are well educated and economically more privileged. Moreover, the percentage of people who reported using digital health technology rose from 70% (1580/2258) to 82.5% (1812/2197) due to the COVID-19 pandemic. This increase was significant for all separate types of digital health technology (all *P*<.001). In addition, we found the interaction effects of COVID-19 with age and education attainment, indicating that the lower total use among older people and people with lower education attainment became slightly less apparent from 2019 to 2020.

**Conclusions:**

These findings on the influence of the COVID-19 pandemic on the digital divide indicated that the use of all types of digital health apps increased and that older individuals and people with a lower level of education caught up a little during COVID-19. Future research should gain more insight into this effect and examine whether it persists beyond the COVID-19 pandemic. Additionally, future endeavors should focus on vulnerable groups, ensuring they receive adequate attention to guarantee access to health care, preventative health-promoting solutions, and social services.

## Introduction

### Background

In recent decades, digital technologies have become increasingly important in the health care sector [1]. The incorporation of digital health technologies is widely recognized as a crucial strategy to address the anticipated health care demand caused by an aging population and a shortage of health care personnel [2,3]. This is a development that international governments gratefully support further through long-term policies and funding [4,5]. Nevertheless, the transition from conventional to digital health care has been accelerated by the impact of the COVID-19 pandemic [6,7]. This poses the risk of creating a “digital divide,” inadvertently placing those who are older, economically disadvantaged, and have a lower level of education at a disadvantage [8-12], raising the question of whether this caused vulnerable groups, with already existing health disparities, to catch up or fall further behind. Therefore, this study focuses on the influence of socioeconomic factors on the adoption of digital health technology in the Frisian population and how this relation is affected by the COVID-19 pandemic.

### Inequalities in Digital Health Technology Adoption

The definition of digital health provided by the World Health Organization (WHO): “the field of knowledge and practice associated with the development and use of digital technologies to improve health” [5], focuses on improving health in the broad sense, going beyond merely applying technology in care contexts by also including preventive health technology in other contexts. For instance, digital health can educate us about health-related symptoms and risks [13,14], support us in the adoption of healthier lifestyles [15,16], and assist us in independent living at older age [3]. This implies that the use of digital technologies is a broad concept.

However, the adoption of digital health technologies has mainly been studied as a narrow notion, predominantly focused on the care context. Digital health technology use was, for instance, operationalized into obtaining health condition information, filling out prescriptions, contacting a clinician, and handling health care insurance matters online and predominantly studied in older people [8], or a combination of patient portal use and health information search behavior on the internet [9]. The results of previous studies indicated that the use of digital technologies, in this narrow sense, was lower in people with older age, lower levels of education, and lower income [8,9,17,18]. Often applied theoretical frameworks for the study of technology acceptance are the technology acceptance model (TAM) and the unified theory of acceptance and use of technology (UTAUT) [19,20]. These models are frequently used to study determinants such as the perceived usefulness of technology and social factors on the intention to use a specific type of technology (group) [21]; whereas, in this study, we focus on socioeconomic factors and a broad operationalization of actual use to examine the digital divide. In conclusion, this study examines the influence of socioeconomic factors on the use of digital health technology in a broader sense, going beyond the care context by also including preventative health-promoting solutions generally available on the consumer market, such as wearables and lifestyle apps, in line with the broad definition of digital health by the WHO.

### Influence of COVID-19

The COVID-19 pandemic accelerated the development of new technologies and contributed significantly to the digitization of various domains of our private and professional lives [22-24], given that the primary behavioral measure used against the pandemic was social distancing [25]. In the health domain, a similar trend was observed, illustrated by the increase in, for instance, the use of online health information or various forms of telemedicine [26].

Policy makers and scientists have wondered whether the global COVID-19 pandemic may have provided circumstances that are all encompassing for even people of older age, with a lower level of education, and with a lower income to catch up [27]. It is also argued that any digital transformation incited by the pandemic will exacerbate preexisting disparities. That is, Crawford and Serhal [28] stated that COVID-19 hits the underprivileged and vulnerable groups harder, and that the development and implementation of digital health solutions within the pandemic response will probably only consolidate the disparities [11]. However, solid empirical grounds for both catching up [29] and falling further behind [26,30] are modest.

This study examined the influence of socioeconomic factors, such as age, income, and educational level, on the self-reported use of digital health technology in the broad sense in the Frisian population and whether this influence was mitigated or intensified by the COVID-19 pandemic.

## Methods

### Study Design

In 2019, prior to the COVID-19 pandemic, and in 2020, during the COVID-19 pandemic, Planbureau Fryslân, the regional office for statistics and policy analyses, and the NHL Stenden University (both organizations in the Netherlands) conducted a panel survey study on digital health in the Frisian population among other survey items regarding other topics. The digital health survey questions were based on preexisting questionnaires on health technology [19,31,32] and developed by experts (n=10) in the field of public health, health technology, regional government development, and epidemiology, leading to consensus about the survey items after 2 iterations. The first iteration of the development of the questionnaire was a face-to-face brainstorming session in September 2019 led by JTBVV and a researcher of Planbureau Fryslân, where a first concept version of the questionnaire was discussed and improved. The second iteration consisted of editorial feedback from the experts on the second concept version of the questionnaire. The questionnaire in 2019 included general health care–related questions (17 questions), use cases on health care technology, and 2 quantitative questions on health care technology. In 2020, several questions from 2019 on digital health were repeated in an omnibus survey on health care and health care technology (8 questions), education and facilities (3 questions), and social challenges (2 questions). The survey question that specifically targeted at the use of digital health apps was included in this study.

The main COVID-19 measures taken by the Dutch government in November 2020 were focused on social distancing [33]. A few examples of the measures taken were the following: (1) a Dutch household could receive up to a maximum of 3 persons per day, (2) cafes and restaurants were closed, (3) outside people were only allowed to be in groups of 4, (4) work from home if possible, and (5) sports activities were only aloud with a maximum of 4 individuals and official matches were prohibited. In hospitals, mouth-nose masks were often obligatory, and most outpatient clinics that had the possibility to provide e-consultations did.

### Participants

A representative sample of about 7000 Frisian (a province in the north of the Netherlands) residents are regularly invited to fill out surveys regarding various topics of regional public concern such as health, economics, and welfare issues by the regional office for statistics and policy analyses. The panel is selected using a stratified random sampling method based on personal characteristics. The panel was invited to fill out the survey items regarding digital health apps for the current study.

### Ethical Considerations

All participants gave ethical permission to participate in the panel and agreed to the use and sharing of data by relevant parties that have a common objective. Participants provided written informed consent upon joining the panel. The panel adheres to the code of conduct established by the Dutch Association for Statistics and Research (*Vereniging voor Statistiek en Onderzoek*). The study is exempt from review by the Ethical Board of NHL Stenden University of Applied Sciences (reference: 202405). The data used for this research were fully anonymized before being shared with the authors of this paper. The authors did not have any contact with the participants, and there was no financial compensation for participating in the panel.

### Procedure

The data were collected by Planbureau Fryslân, who sent out Dutch web-based surveys (Quest Software, Inc) by email to their panel. Panel members received 2 reminder emails and were given 1 month to complete the survey.

### Survey Items

To quantify the use of digital health technologies, the following survey item was used: “Which of the following digital health apps do you currently use?” The response options included 4 items on general health communication and information (CI): text messaging to family about shared parenting or informal care, platform or app for social contact with others, app with health information, and online health information; 3 items on patient-provider communication (PPC): patient portal, scheduling an appointment with general practitioner online, and e-consultations; and 6 items on proactive health behavior (PHB): lifestyle apps, a wearable, home-assistant, platform or app for offering practical assistance, domotics, personal alarm or fall detection sensors, and none of these. The categorization was derived from a number of earlier studies [17,31,32,34]. The use of digital health apps was quantified using the sum of options that were ticked except for none of these, which was quantified as 0, resulting in an ordinal variable ranging from 0 to 13. This item was collected twice; once in October 2019 and once in November 2020.

The sociodemographic characteristic variables education attainment (low: less than primary, primary, and lower secondary education; medium: upper secondary and postsecondary nontertiary education; and high: tertiary education, ie, education provided by universities and other higher education institutions), self-rated perceived health (very discontent, discontent, nor content or discontent, content, and very content), and income (below modal <€36,500 [US $40,560], modal: €36,500 to €43,500 [US $40,560 to US $48,338], and above modal >€43,500 [US $48,338]) were collected in the enquiry in 2019. The variables sex (male or female), age (collected in ranges of 5 years; recoded into 18-49 years, 50-74 years, or 75 years and older), and residential context (urban>15,000 or rural<15,000) were collected upon registration for the panel.

### Statistical Analysis

Descriptive statistics are reported using frequencies, percentages, median, and IQR. A Kendall τ_b_ correlation was run to determine the relationships between the ordinal socioeconomic factors, where a correlation from 0.1 to 0.4 was interpreted as weak, from 0.4 to 0.7 as moderate, and >0.7 as strong [35]. The statistical analyses addressed 3 topics. First, to assess the influence of socioeconomic factors on the total use of digital health apps, a generalized linear model was fitted with the use of digital health apps as the dependent variable and socioeconomic factors as between-subject factors on the 2019 data. Second, to analyze whether the use of separate health apps increased from 2019 to 2020, we conducted chi-square tests on the different survey options. Third, to examine the influence of COVID-19 on the use of digital health apps, a generalized linear mixed model was fitted with use of digital health app as the dependent variable, COVID-19 as the within-subject variable and socioeconomic factors as between-subject factors. As the dependent variable “use of digital health apps” was a count variable, all mixed models were fitted with a Poisson distribution. Furthermore, all models were run with and without the socioeconomic characteristic of income, as this factor was often not reported. Tukey post hoc tests on perceived health or contrasts in age, education attainment, and income were performed if the main effects were significant. The generalized linear mixed models were performed in R (version 4.3.1, R Foundation for Statistical Computing) using the *lme4* (version 1.1-35.1) toolbox [36]. *P* values and other statistics of the models were computed using the *car* package (version 3.1-2). If a model failed to converge, the *bobyqa* optimizer was applied. The chi-square tests and correlation analyses were performed in SPSS (version 27.00, IBM Corp). The significance level was set at α=.05.

## Results

### Sociodemographic Characteristics

The sociodemographic characteristics of the 2258 participants are reported in Table 1. The age range of all participants (n=845, 37.4% female) was between 20 and 99 years, with a large number of people aged >65 years (n=1417, 62.8%). A small majority (n=1164, 51.6%) lived in urban areas, while 48.4% (n=1049) lived in rural settings. The education attainment was low to middle in 49.4% (n=1115) of the population, and almost half (416/883, 47.1%) of the population reported above modal income. However, 60.9% (1375/2258) of the panel chose not to report income levels. The vast majority (n=2072, 91.8%) of the population was to some extent content to very content with their perceived health. There was a weak, positive correlation between income level and educational attainment (τ_b_=0.14; *P*<.001) and a weak, negative correlation between income level and age (τ_b_=–0.12; *P*<.001).

**Table 1:** Descriptive statistics on the sociodemographic characteristics (sex, age, residential context, education attainment, income, and perceived health) of the Frisian survey panel (N=2258).

Variables		Values, n (%)
**Sex**		
	Male	1413 (62.6)
	Female	845 (37.4)
**Age group (years)**		
	18-49	200 (8.8)
	50-64	641 (28.4)
	65-79	1199 (53.1)
	80 and older	218 (9.7)
**Residential context**		
	Rural	1094 (48.4)
	City	1164 (51.6)
**Educational attainment**		
	Low	448 (19.8)
	Middle	667 (29.5)
	High	1143 (50.6)
**Income**		
	Below modal level	340 (15.1)
	Modal level	127 (5.6)
	Above modal level	416 (18.4)
	Missing	1375 (60.9)
**Perceived health**		
	Very discontent	68 (3.0)
	Discontent	118 (5.2)
	Nor content nor discontent	323 (14.3)
	Content	1143 (50.6)
	Very content	598 (26.5)
	Missing	8 (<1.0)

### Influence of Socioeconomic Factors on the use of Digital Health Technology in 2019

In 2019, before COVID-19, a total of 70% (n=1580) of the population used 1 or more forms of health technology health technology (range: 1-10), whereas the majority (n=1207, 53.5%) reported using 1 to 3 health technologies.

**Figure 1:**
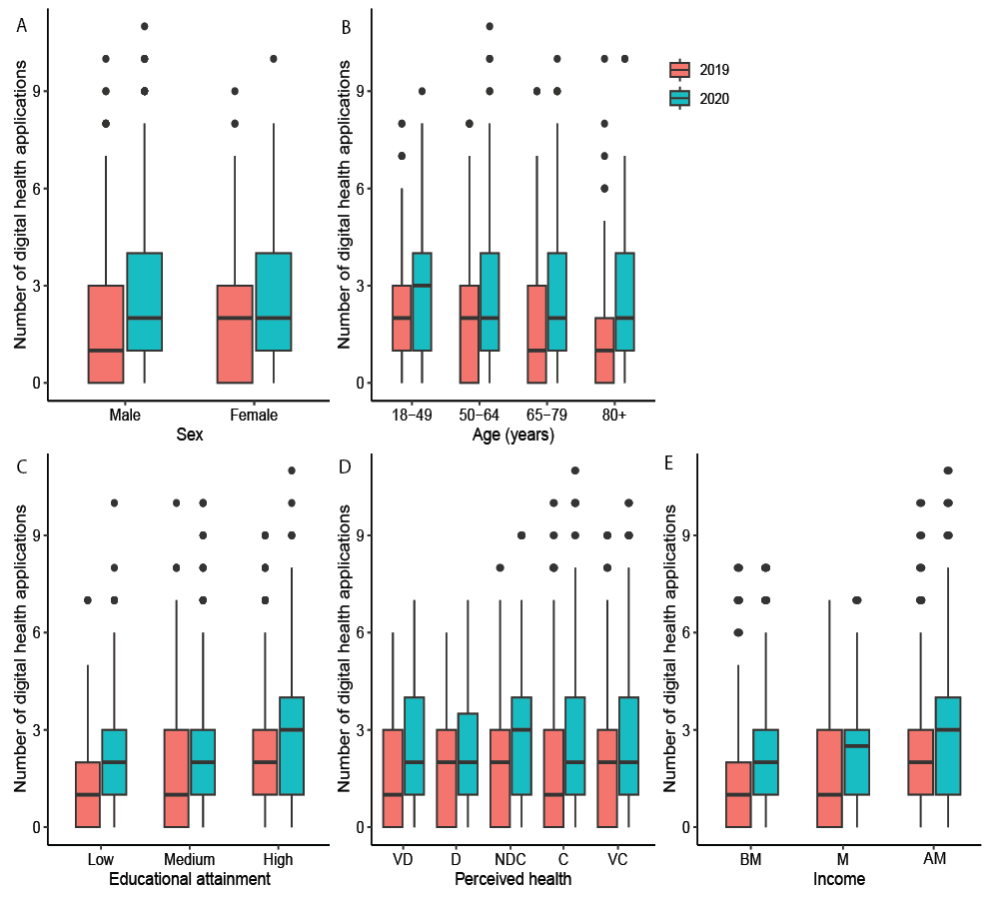
Boxplot of influence of socioeconomic factors and COVID-19 on the use of digital health apps in the Frisian survey panel. The red boxplots represent the number of digital health apps used prior to COVID-19 (2019) and the blue boxplots represent the number of digital health apps used during COVID-19 (2020). The boxplots include the median (fat line), the IQR (box), and the outliers (dots). The number of digital health apps indicates the total amount of apps used. (A) The relationship between use and sex for prior to COVID-19 (2019) and during COVID-19 (2020); (B) the relationship between use and age in 2019 and 2020; (C) the relation between use and education attainment in 2019 and 2020; and (D) the relation between use and perceived health in 2019 and 2020.; and (E) the relation between use and income in 2019 and 2020. Note that income is presented with n=833 and all other variables with n=2157. AM: above modal; BM: below modal; C: content; D: discontent; M: modal; NDC: neither discontent nor content; VC: very content; VD: very discontent.

First, we analyzed whether the socioeconomic factors (sex, age, education attainment, perceived health, and residential context) affected the use of digital health technology. There were significant main effects of sex (*χ*^2^_1_=7.24, N=2197, *P*=.007), age (*χ*^2^_3_=18.58, N=2197, *P*=.003), education attainment (*χ*^2^_2_=147.3, N=2197, *P*<.001), and perceived health (*χ*^2^_4_=10.5, N=2197, *P*=.03). The main effect of sex indicated that the use was higher among women compared to men (Figure 1A, red boxplots). The post hoc linear contrast of age (*z*=–3.63; *P*<.001) indicated that the use was lower in people of older age (Figure 1B, red boxplots). The post hoc linear contrast of education attainment (*z*=11.00; *P*<.001) indicated that the use was higher in people with higher levels of education (Figure 1C, red boxplots). The Tukey post hoc test of perceived health revealed 1 significant effect indicating that use is higher in people who are neither content nor discontent compared to people who are content with their perceived health (*z*=2.8; *P*=.03; Figure 1D, red boxplots).

Second, when including income in the analysis, the number of observations decreases to 883. In this analysis, the main effects of sex (*χ*^2^_1_=12.7, n=883, *P*<.001), age (*χ*^2^_3_=18.8, n=883, *P*<.001), education attainment (*χ*^2^_2_=9.7, n=883, *P*=.008), and perceived health (*χ*^2^_4_=18.9, n=883, *P*<.001) were similar to the previous analysis. Furthermore, we found the effect of income (*χ*^2^_2_=40.0, n=883, *P*<.001). The post hoc linear contrast on income (*z*=–3.6; *P*<.001) indicated that the use was higher in people with a higher income (Figure 1F, red boxplots).

### Influence of COVID-19 on the use of Digital Health Technology

Of the 70% (n=1580) of the population that used one or more forms of health technology in 2019, the most commonly used form was searching for health information on the internet, whereas the use of text messaging, patient portals, platforms or apps for social contact with others, and scheduling appointments online are used by at least one-fifth of the research population (Table 2). Moreover, the category general health communication and information is used most often. Wearables and lifestyle apps are used by 9.4% (n=213) and 16.2% (n=366) of the population, respectively. Technologies that specifically support ambient assisted living, such as a home assistant, domotics, and personal alarm systems are less commonly used, resulting in the least usage in the proactive health behavior category. This was also the case for e-consults, platforms, or apps for offering practical assistance.

In 2020, during the COVID-19 pandemic, the number of reported users increased to 82.5% (n=1812; *χ*^2^_1_=208.3, N=2197, *P*<.001). As depicted in Table 2, the use of all digital health technologies increased from 2019 to 2020. Finding health information on the internet remained an important type of use, although it did not increase much from 2019 to 2020. The use of text messaging, patient portals, platforms or apps for social contact with others, and scheduling appointments online increased to about 30% of the research population. In addition, a considerable increase was noticed in e-consultations (n=28, 1.2% to n=140, 6.4%).

**Table 2:** Descriptive statistics on the use of digital health technologies in the Frisian survey panel. The types of digital health technologies are organized into 3 categories: CI^a^, PPC^b^, and PHB^c^. In addition, the chi-square test and *P* values are provided for each type of technology testing the increase in use from 2019 to 2020.

Category and type of technology			2019 (N=2258), n (%)		2020 (n=2197), n (%)		Chi-square (*df*; n=2197)		*P* value
**CI**									
	Text messaging to family about shared parenting or informal care	552 (24.4)		787 (35.8)		220.1 (1)		<.001	
	Platform or app for social contact with others	485 (21.5)		749 (34.1)		244.3 (1)		<.001	
	Health information on the internet	1065 (47.2)		1130 (51.4)		343.6 (1)		<.001	
	An app with health information	89 (3.9)		147 (6.7)		158.2 (1)		<.001	
**PPC**									
	Patient portals	524 (23.2)		783 (35.6)		338 (1)		<.001	
	Scheduling an appointment with general practitioner online	474 (21)		612 (27.9)		340 (1)		<.001	
	E-consultations	28 (1.2)		140 (6.4)		99.4 (1)		<.001	
**PHB**									
	Lifestyle app	366 (16.2)		515 (23.4)		511.9 (1)		<.001	
	Wearable	213 (9.4)		268 (12.2)		422.1 (1)		<.001	
	Home assistant	86 (3.8)		153 (7)		219.4 (1)		<.001	
	Platform or app for offering practical assistance	29 (1.3)		44 (2)		38 (1)		<.001	
	Domotics	113 (5)		161 (7.3)		564.4 (1)		<.001	
	Personal alarm system or fall detection with sensors	31 (1.4)		40 (1.8)		151.9 (1)		<.001	

^a^CI: general health communication and information.

^b^PPC: patient-provider communication.

^c^PHB: proactive health behavior.

### Interaction of Socioeconomic Factors and COVID-19 on the use of Digital Health Technology

From 2019 to 2020, the part of the population that used a form of health technology increased from 70% (n=1580) to 82.5% (n=1812; *χ*^2^_1_=208.3, N=2197, *P*<.001). First, we assessed if the change in the use of digital health technology due to the COVID-19 pandemic interacted with any socioeconomic factors (sex, age, education attainment, and perceived health). Note that residential context was left out of the analysis as this factor was not significant in the prior analysis. The main effects of COVID-19 (*χ*^2^_1_=5.2, N=2157, *P*=.02), sex (*χ*^2^_1_=7.0, N=2157, *P*=.008), age (*χ*^2^_3_=11.2, N=2157, *P*=.01), and education attainment (*χ*^2^_2_=99.1, N=2157, *P*<.001) were rather similar to the 2019 analysis above. There were interaction effects of COVID-19 with age (*χ*^2^_3_=15.7, N=2157, *P*<.001) and education attainment (*χ*^2^_2_=10.8, N=2157, *P*=.004). The interaction effect of age indicated that the lower use among people in older age categories became less apparent from 2019 to 2020 (Figures 1A and 1B). The interaction effect of education attainment indicated that the lower use in people with a low educational level became less apparent from 2019 to 2020. Upon closer inspection, the health technologies classified as CI (Table 2) seem to be primarily responsible for these effects (Multimedia Appendix 1), as in the categories PPC and PHB, only main effects were found. One specific interesting finding using the classification is that PPC (Table 2) is not related to age (Multimedia Appendix 1).

Second, when income is included in the analysis, the main effects of sex (*χ*^2^_1_=10.0, n=883, *P*=.02), age (*χ*^2^_3_=9.7, n=883, *P*=.01), education attainment (*χ*^2^_2_=7.1, n=883, *P*=.03), perceived health (*χ*^2^_4_=12.8, n=883, *P*=.01), and income (*χ*^2^_2_=27.3, n=883, *P*<.001) were rather similar to the 2019 analysis above. The interaction effect of COVID-19 and education attainment is not replicated in this smaller cohort, but the interaction effect of COVID-19 and age (*χ*^2^_3_=12.1, n=883, *P*=.006) remained. No interaction effect of COVID-19 and income was observed.

## Discussion

### Principal Findings

This study examined the influence of socioeconomic factors, such as age, income, and educational attainment, on the use of digital health technology in the Frisian population and whether this was mediated by COVID-19. Self-reported use of digital health technology was investigated in the broader sense, going beyond the care context, by also including preventative health-promoting solutions generally available on the consumer market, such as wearables and lifestyle apps. Our results indicate that prior to the COVID-19 pandemic, digital health technology use was higher in women, younger people, and those who are well educated and economically more privileged. Furthermore, there was a significant increase in all types of digital health apps from before to during COVID-19. Additionally, our findings on the influence of the COVID-19 pandemic on the digital divide indicated that older persons and people with a lower level of education caught up a little during COVID-19.

The dataset in this study provided for the possibility to study how the COVID-19 pandemic and socioeconomic factors interact with regard to the adoption of digital health technologies, showing that the digital divide seemed to decrease a little and older individuals and people with low levels of education somewhat caught up on use. To our knowledge, this panel study prior to and during COVID-19 with a broad operationalization of digital health technologies use is novel. However, how the COVID-19 pandemic and socioeconomic factors interact with regard to patient portal use has been investigated in earlier research, indicating that racial differences in use decreased during the pandemic [29]. In addition, a cross-sectional study on the self-rated increase in internet use for health and social services due to COVID-19 revealed that only education attainment was associated with increased use, while other variables, such as age, sex, and income, were not related to a change in use [30]. Our results are in line with these findings and concentrate on older individuals and people with lower levels of education, demonstrating that there is growing evidence that the all-encompassing circumstances of COVID-19 provided the possibility to decrease the digital divide a little. Nevertheless, the increase in use did not concern all socioeconomic factors, suggesting that although the digital divide seems to decrease a little, vulnerable populations still require special attention, especially in today’s rapidly digitizing society.

When examining the separate digital health apps more closely, the use of all items increased during COVID-19. The most common form of digital health technology was searching for health information on the internet. This form of health technology has been extensively researched separately, showing that older people with low socioeconomic status are less likely to use the internet to look for health information [9,17,18,37]. In the current cohort, the use of text messaging, patient portals, platforms, or apps for social interactions with others and scheduling appointments online increased up to about one-third of the population due to COVID-19. Moreover, technologies from the category CI were used most often. In addition, PHB, such as the use of wearables and lifestyle apps, were adopted the least, by up to a quarter of the population during COVID-19. Our different operationalization seems to identify similar socioeconomic factors affecting digital health technology use as shown in earlier research [9,17,18,37]. Note that all related studies mentioned in the previous sections report changes in quantity of use and not duration or frequency of use.

Our results also show that women, people with high educational levels, young individuals, and wealthy individuals are more likely to use digital health technologies. These effects of sex [9,17], education attainment, age, and income replicate the results found in other studies [9,17,18,37]. Furthermore, rather similar influencing socioeconomic factors have been identified in research into eHealth literacy [11,38]. Additionally, previous research on the relationship between perceived health and health technology use has shown contrasting effects. That is, if perceived health is poor, digital health care technology use is lower [8] and the use of health information on the internet is lower [39], whereas electronic patient record use is higher if perceived health is poorer [34]. In this study, post hoc tests only revealed 1 small difference in digital health technology use between groups that perceived their health as relatively content. One possible explanation for our results could be that we defined the use of digital health apps broader than in earlier studies, which also reported opposite results for different kinds of technologies.

The findings of our study can to some extent be related to the theoretical technology acceptance framework UTAUT [19]. For example, in the UTAUT model, the 2 demographic variables of the study, age and sex, are incorporated as moderating factors on (intended) use. Our results indicated that during COVID-19 age became a slightly less dividing factor, with people of older age catching up a little on self-reported technology use. How the factor of age influences self-reported use, as described in the UTAUT model, was beyond the scope of this study. Additionally, our outcomes are not in line with the expectations on intended use by a study in another Dutch region among older adults prior and during the COVID-19 pandemic using the TAM [21]. This study indicated that the intention of older people to use a specific mHealth app had not changed during COVID-19 compared to prior to COVID-19, whereas our study indicated that older persons did use slightly more digital health technologies during the COVID-19 pandemic, compared to before. An explanation for this could be that the use of 1 specific app is less susceptible to change by an external factor such as COVID-19.

For the generalizability of the current findings, one should be able to consider the demographic characteristics of Fryslân with regard to the Netherlands and Europe. The province of Fryslân is considered a rural area according to Dutch standards and is currently facing an ageing society and a decrease in the general population [40]. These trends are comparable to other rural areas in Europe.

### Limitations

The study contains a large cohort with 2258 respondents. However, there was a selection bias in the cohort. Whereas a representative panel was invited to fill out the questionnaire, individuals with lower income, those with lower educational attainment, younger people, and female individuals had lower response rates. When zooming in on one of these factors, we see that older people are overrepresented in the cohort, with 63% of the cohort being aged 65 years or older. While this overrepresentation of older people occurred, we still found an age effect indicating that their use was lower, and they caught up a little on the use of digital health technologies during COVID-19. Additionally, the number of male respondents was much higher than female respondents. Both the overrepresentation of older people and male individuals were due to selection bias, as the panel is a representative selection of the Frisian population where about 50% of the population is male and 75% of the people are younger than 65 years [40].

Furthermore, the number of missing values in the socioeconomic variable income might have led to a selection bias. Recipients who did respond to this variable might have a higher income than people who did not respond, which might have influenced the results. The selection biases on income and age might also explain the unexpectedly weak, negative correlation between income and age. Moreover, this study focused on the type of technology use operationalized into 1 specific tick-box question and did not include depth of use related to duration and frequency, which could provide a more extensive description of health technology use. Finally, all recipients demonstrated selection bias due to the digital nature of the questionnaire and their interest in filling out a questionnaire on digital health, indicating that the use in the general population is likely to be lower.

Understanding whether and how being infected with COVID-19 affected participants’ health app use patterns would also have been interesting, especially related to our findings regarding senior citizens. Older people are more prone to suffer from severe COVID-19 [41,42], which could potentially explain the increase in health technology use, specifically for PPC. However, upon closer inspection, our results on the 3 separate categories of apps indicate that PPC is not related to age, suggesting that the higher COVID-19 risk for senior citizens is not a confounding factor in our analyses.

### Future Research

This study examined the digital divide in health technology use and found that it was mildly reduced for educational attainment and age during the COVID-19 pandemic. Given the extreme all-encompassing scenario of COVID-19, future research should focus on whether this mitigation of the digital divide persists beyond the pandemic. Moreover, they should include frequency and duration of use in addition to self-reported use only and might also include quantitative data on, for instance, patient portal use, to gain more in-depth insight into technology use. Taken together, this will provide a deeper understanding on how changes in the digital divide impact the long-term use of health technologies. In addition, a mixed-design methodology might be considered, where 1 type of health technology is examined in more detail focusing on engagement with [43] and acceptance of [19] health technology.

In addition, future research should include an eHealth literacy scale [31] to examine the relation between eHealth literacy and digital health technology use in the broad sense. Previous research indicated a positive relation between health literacy and health-related information seeking on the internet [44,45]. It is currently unclear whether this also holds for eHealth literacy and digital health technology use as defined in the broad sense in this paper, going beyond just online health-related information seeking on the internet. This could help reveal specific types of digital health technologies that are associated with lower eHealth literacy and guide interventions to decrease the digital divide. Furthermore, the results of the study suggest that specific interventions should be targeted at people with a low socioeconomic status to ensure proper access to health care, social services, and preventive health promotion solutions for these vulnerable groups. Targeted interventions should address this with community-based cocreation methodologies applying human-centered design [46-48]. Some recent pilot projects in several Frisian communities seem to indicate that informal initiatives in villages and neighborhoods are important hubs in the strategy to increase digital health technology use and eHealth literacy in these communities. These interventions are in line with long-term policies and funding focused on digital health technology [4,5].

### Conclusions

This study examined the influence of socioeconomic factors, such as age, income, and educational attainment, and the COVID-19 pandemic on digital health technology use in the Frisian population. Our results indicated that digital health technology use in 2019 was higher in women, younger individuals, and those with higher incomes and higher educational attainment. Furthermore, the use of all types of digital health apps increased during COVID-19 compared to before. Additionally, during the COVID-19 pandemic older people and those with lower levels of education caught up a little on digital health app use when compared to before COVID-19. Future research should gain more insight into this effect and focus on whether this effect persists after COVID-19 and on vulnerable groups that still require special attention to ensure proper access to health care, social services, and preventive health promotion solutions for these vulnerable groups.

## Appendix

Multimedia Appendix 1 Additional analyses on the categories of health technologies.

## Abbreviations

CI: general health communication and information
PPC: patient-provider communication
PHB: proactive health behavior
TAM: technology acceptance model
UTAUT: unified theory of acceptance and use of technology
WHO: World Health Organization

## Data Availability

The data used in this research are available upon request, provided that the request aligns with the established conditions, which can be found at www.planbureaufryslan.nl. Researchers interested in using these data should contact the Planbureau Fryslân Office at info@planbureaufryslan.nl.

## References

[1] Bhavnani SP, Narula J, Sengupta PP (2016). Mobile technology and the digitization of healthcare. Eur Heart J.

[2] Taj F, Klein MCA, van Halteren A (2019). Digital health behavior change technology: bibliometric and scoping review of two decades of research. JMIR Mhealth Uhealth.

[3] Chen C, Ding S, Wang J (2023). Digital health for aging populations. Nat Med.

[4] (2022). eHealth. Shaping Europe's Digital Future.

[5] (2021). Global strategy on digital health 2020-2025. World Health Organization.

[6] Keesara S, Jonas A, Schulman K (2020). Covid-19 and health care's digital revolution. N Engl J Med.

[7] Golinelli D, Boetto E, Carullo G, Nuzzolese AG, Landini MP, Fantini MP (2020). Adoption of digital technologies in health care during the COVID-19 pandemic: systematic review of early scientific literature. J Med Internet Res.

[8] Levine DM, Lipsitz SR, Linder JA (2016). Trends in seniors' use of digital health technology in the United States, 2011-2014. JAMA.

[9] Mahajan S, Lu Y, Spatz ES, Nasir K, Krumholz HM (2021). Trends and predictors of use of digital health technology in the United States. Am J Med.

[10] van Deursen AJAM, van Dijk JAGM (2011). Internet skills performance tests: Are people ready for eHealth?. J Med Internet Res.

[11] de Santis KK, Jahnel T, Sina E, Wienert J, Zeeb H (2021). Digitization and health in Germany: cross-sectional nationwide survey. JMIR Public Health Surveill.

[12] Yao R, Zhang W, Evans R, Cao G, Rui T, Shen L (2022). Inequities in health care services caused by the adoption of digital health technologies: scoping review. J Med Internet Res.

[13] Win KT, Hassan NM, Bonney A, Iverson D (2015). Benefits of online health education: perception from consumers and health professionals. J Med Syst.

[14] Zhao J, Han H, Zhong B, Xie W, Chen Y, Zhi M (2021). Health information on social media helps mitigate Crohn's disease symptoms and improves patients' clinical course. Comput Hum Behav.

[15] Chatterjee A, Prinz A, Gerdes M, Martinez S (2021). Digital interventions on healthy lifestyle management: systematic review. J Med Internet Res.

[16] Thomas JG, Bond DS (2014). Review of innovations in digital health technology to promote weight control. Curr Diab Rep.

[17] Calixte R, Rivera A, Oridota O, Beauchamp W, Camacho-Rivera M (2020). Social and demographic patterns of health-related internet use among adults in the United States: a secondary data analysis of the Health Information National Trends Survey. Int J Environ Res Public Health.

[18] Hong YA, Cho J (2017). Has the digital health divide widened? Trends of health-related internet use among older adults from 2003 to 2011. J Gerontol B Psychol Sci Soc Sci.

[19] Venkatesh V, Morris MG, Davis GB, Davis FD (2003). User acceptance of information technology: toward a unified view. MIS Q.

[20] Rouidi M, Elouadi AE, Hamdoune A, Choujtani K, Chati A (2022). TAM-UTAUT and the acceptance of remote healthcare technologies by healthcare professionals: a systematic review. Inform Med Unlocked.

[21] van Elburg FRT, van de Klundert J, Nieboer AP, Askari M (2023). The intention to use mHealth applications among Dutch older adults prior and during the COVID pandemic. Front Public Health.

[22] Almeida F, Duarte Santos J, Augusto Monteiro J (2020). The challenges and opportunities in the digitalization of companies in a post-COVID-19 world. IEEE Eng Manag Rev.

[23] Chan XW, Shang S, Brough P, Wilkinson A, Lu C (2022). Work, life and COVID-19: a rapid review and practical recommendations for the post-pandemic workplace. Asia Pac J Human Res.

[24] Cone L, Brøgger K, Berghmans M, Decuypere M, Förschler A, Grimaldi E, Hartong S, Hillman T, Ideland M, Landri P, van de Oudeweetering K, Player-Koro C, Bergviken Rensfeldt A, Rönnberg L, Taglietti D, Vanermen L (2021). Pandemic acceleration: COVID-19 and the emergency digitalization of European education. Eur Educ Res J.

[25] Nande A, Adlam B, Sheen J, Levy MZ, Hill AL (2021). Dynamics of COVID-19 under social distancing measures are driven by transmission network structure. PLoS Comput Biol.

[26] Zhou W, Cho Y, Shang S, Jiang Y (2023). Use of digital health technology among older adults with cancer in the United States: findings from a national longitudinal cohort study (2015-2021). J Med Internet Res.

[27] Torous J, Jän Myrick K, Rauseo-Ricupero N, Firth J (2020). Digital mental health and COVID-19: using technology today to accelerate the curve on access and quality tomorrow. JMIR Ment Health.

[28] Crawford A, Serhal E (2020). Digital health equity and COVID-19: the innovation curve cannot reinforce the social gradient of health. J Med Internet Res.

[29] Mai F, Ko DG, Shan Z, Zhang D (2023). The impact of accelerated digitization on patient portal use by underprivileged racial minority groups during COVID-19: longitudinal study. J Med Internet Res.

[30] Heponiemi T, Virtanen L, Kaihlanen AM, Kainiemi Päivikki Koponen E, Koskinen S (2022). Use and changes in the use of the internet for obtaining services among older adults during the COVID-19 pandemic: a longitudinal population-based survey study. New Media Soc.

[31] van der Vaart R, Drossaert C (2017). Development of the digital health literacy instrument: measuring a broad spectrum of health 1.0 and health 2.0 skills. J Med Internet Res.

[32] Paige SR, Stellefson M, Krieger JL, Anderson-Lewis C, Cheong J, Stopka C (2018). Proposing a transactional model of eHealth literacy: concept analysis. J Med Internet Res.

[33] (2020). November 2020: increase and extension of partial lockdown [Article in Dutch]. Rijksoverheid.

[34] Zheng H, Jiang S (2022). Frequent and diverse use of electronic health records in the United States: a trend analysis of national surveys. Digit Health.

[35] Dancey CP, Reidy J (2007). Statistics Without Maths for Psychology.

[36] Bates D, Mächler M, Bolker B, Walker S (2015). Fitting linear mixed-effects models using lme4. J Stat Soft.

[37] Jacobs W, Amuta AO, Jeon KC (2017). Health information seeking in the digital age: an analysis of health information seeking behavior among US adults. Cogent Soc Sci.

[38] Holt KA, Overgaard D, Engel LV, Kayser L (2020). Health literacy, digital literacy and eHealth literacy in Danish nursing students at entry and graduate level: a cross sectional study. BMC Nurs.

[39] Bounsanga J, Voss MW, Crum AB, Hung M (2016). The association between perceived health status and health information communication channels. J Health Commun.

[40] CBS Open Data StatLine.

[41] Gao YD, Ding M, Dong X, Zhang JJ, Kursat Azkur A, Azkur D, Gan H, Sun YL, Fu W, Li W, Liang HL, Cao YY, Yan Q, Cao C, Gao HY, Brüggen MC, van de Veen W, Sokolowska M, Akdis M, Akdis CA (2021). Risk factors for severe and critically ill COVID-19 patients: a review. Allergy.

[42] Ou M, Zhu J, Ji P, Li H, Zhong Z, Li B, Pang J, Zhang J, Zheng X (2020). Risk factors of severe cases with COVID-19: a meta-analysis. Epidemiol Infect.

[43] Kelders SM, Kip H (2019). Development and initial validation of a scale to measure engagement with eHealth technologies.

[44] Lee HY, Jin SW, Henning-Smith C, Lee J, Lee J (2021). Role of health literacy in health-related information-seeking behavior online: cross-sectional study. J Med Internet Res.

[45] Estacio EV, Whittle R, Protheroe J (2019). The digital divide: examining socio-demographic factors associated with health literacy, access and use of internet to seek health information. J Health Psychol.

[46] Faber JS, Al-Dhahir I, Reijnders T, Chavannes NH, Evers AWM, Kraal JJ, van den Berg-Emons HJG, Visch VT (2021). Attitudes toward health, healthcare, and eHealth of people with a low socioeconomic status: a community-based participatory approach. Front Digit Health.

[47] van Leersum CM, Konrad KE, Siebrand E, Malik ZB, den Ouden MEM, Bults M (2023). Engaging older adults with a migration background to explore the usage of digital technologies in coping with dementia. Front Public Health.

[48] Batterham RW, Buchbinder R, Beauchamp A, Dodson S, Elsworth GR, Osborne RH (2014). The OPtimising HEalth LIterAcy (Ophelia) process: study protocol for using health literacy profiling and community engagement to create and implement health reform. BMC Public Health.

