# Supplementary material for: Digital Health Technology Use Across Socioeconomic Groups Prior to and During the COVID-19 Pandemic: Panel Study
Source: JMIR Public Health Surveill. 2024 Sep 13;10:e55384. doi: 10.2196/55384 (PMC11437226; doi:10.2196/55384)
Supplement: Multimedia Appendix 1 [file publichealth_v10i1e55384_app1.docx]

**Supplementary material**

This document contains additional analyses on the categories of health technologies. The three categories are general health communication and information (CI), patient-provider communication (PPC), and proactive health behavior (PHB), containing the following health technologies: text messaging to family about shared parenting or informal care, platform or app for social contact with others, app with health information, and online health information for CI; patient portal, scheduling an appointment with general practitioner online, and e-consultations for PPC; and lifestyle apps, an a wearable, a home-assistant, platform or app for offering practical assistance, domotics, and personal alarm or fall detection sensors for PHB. To examine the influence of COVID-19 on the use of categories of digital health applications three generalized linear mixed model were fitted with use of CI, PCC, and PHB as dependent variables, COVID-19 as within subjects variable, and age and education attainment (as those showed significant interaction effects in the analyses in the paper) as between subject factors. Post-hoc contrast analyses on main effects were performed when appropriate.

For the analyses on CI, there were significant main effects of COVID-19 (χ2 (1, N=2197) = 4.55, P = 0.03), age (χ2 (3, N=2197) = 12.36, P = 0.006), and education attainment (χ2 (2, N=2197) = 72.21, P < 0.001). There were interaction effects of COVID-19 with age (χ^2^(3, N=2157) = 13.03, *P* = 0.004) and education attainment (χ^2^(2, N=2157) = 11.43, *P* = 0.003). The interaction effect of age indicated that the lower use of CI among people in older age categories became less apparent from 2019 to 2020 (see figure 1 panel A and B). The interaction effect of education attainment indicated that the lower use in low education attainment became less apparent from 2019 to 2020. The category general health communication and information replicated the effects of the main analyses in the paper.

Concerning patient-provider communication, there was a significant main effect of education attainment (χ2 (2, N=2197) = 33.77, P < 0.001). The post-hoc linear contrast of education attainment (z = 6.48, *P* <0.001) indicated that use was higher in people with a higher education (see figure 1 panel C and D). The patient-provider communication category did not replicate all effects of the main analyses, indicating that for this type of health technology the digital divide did not seem to decrease.

The proactive health behavior category also did not replicate all effects of the main analyses. There were significant main effects of COVID-19 (χ2 (1, N=2197) = 5.58, P = 0.02), age (χ2 (3, N=2197) = 23.72, P < 0.001), and education attainment (χ2 (2, N=2197) = 21.52, P < 0.001). The post-hoc linear contrast of age (z = -4.01, *P* <0.001) indicated that use was lower in the elderly (see figure 1 panel E and F). The post-hoc linear contrast of education attainment (z = 5.01, *P* <0.001) indicated that use was higher in people with a higher education (see figure 1). When taking a closer look at figure 1, it shows that the median of proactive health behavior is 0 for all education attainment levels and age levels, so the use of preventative technological solutions was low.

Taken together, these analyses could indicate that the main effects in the paper are mainly driven by the general health communication and information category. However, note that these additional analyses should be taken with caution as the number of possible options in especially the category patient-provider communication is low for a Poisson distribution. This could explain why the effects of the main analyses in the paper were not replicated in this category, while figure 1 suggests a trend towards the main outcomes of the paper. Additionally, the median of the proactive health behavior category was low and contained most outliers. A last note of caution is that in general these analyses involve double dipping by performing additional statistical analyses on a specific parts of the data, while other post-hoc tests have already been performed.

**Figure S1**

**
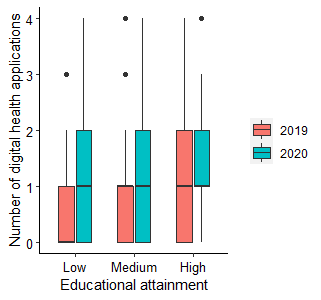
A** CI **B** CI

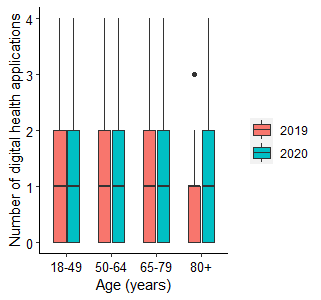


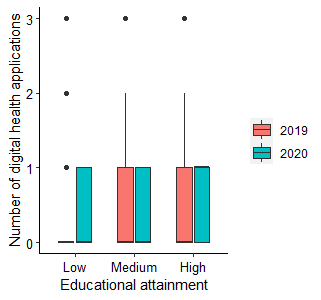
**C** PPC **D** PPC

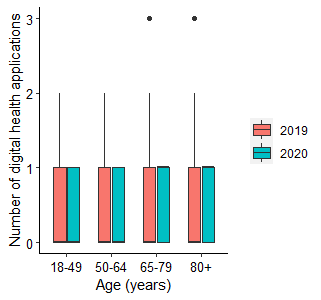


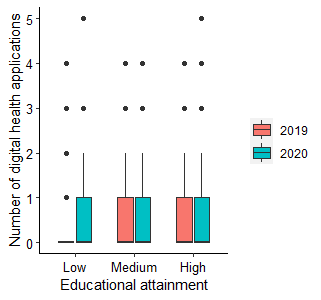
**E** PHB **F** PHB

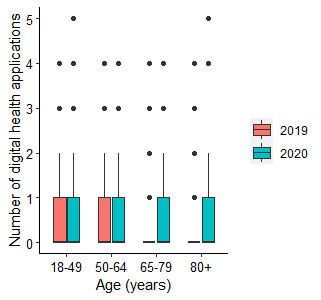


Figure S1. *Boxplot of influence of age, educational attainment, and COVID-19 on the use of digital health applications.*

Panel A and B show the effects of age and education attainment on the category general health communication and information. Panel C and D show the effects of age and education attainment on the category patient-provider communication. Panel E and F show the effects of age and education attainment on the category proactive health behavior. The circles display the outliers.
